# Improvements of Age-Related Cognitive Decline in Mice by *Lactobacillus helveticus* WHH1889, a Novel Strain with Psychobiotic Properties

**DOI:** 10.3390/nu15173852

**Published:** 2023-09-03

**Authors:** Kan Gao, Cailing Chen, Xueqin Ke, Qiuling Fan, Haifeng Wang, Yanjun Li, Su Chen

**Affiliations:** 1 Research and Development Department, Hangzhou Wahaha Group Co., Ltd., Hangzhou 310018, China; kevingogh911@hotmail.com (K.G.); chencailing@wahaha.com.cn (C.C.); xueqin.ke@wahaha.com.cn (X.K.); qiuling.fan@wahaha.com.cn (Q.F.); lyj@wahaha.com.cn (Y.L.); 2 Key Laboratory of Food and Biological Engineering of Zhejiang Province, Hangzhou 310018, China; 3 MOE Key Laboratory of Molecular Animal Nutrition, College of Animal Science, Zhejiang University, Hangzhou 310058, China; 4 College of Biosystems Engineering and Food Science, Zhejiang University, Hangzhou 310058, China

**Keywords:** gut microbiota–brain axis, age-related cognitive decline, *Lactobacillus helveticus*, 5-HTP, gut microbiome

## Abstract

A gradual decline in cognitive function occurs with age. Accumulating evidence suggests that certain probiotic strains exert beneficial effects on age-related cognitive decline. Our previous study revealed that *Lactobacillus helveticus* WHH1889 attenuated symptoms of anxiety and depression in depressed mice via shaping the 5-hydroxytryptamine (5-HT) and 5-hydroxytryptophan (5-HTP) metabolism and gut microbial community, indicating the psychobiotic potential of WHH1889. In the present study, the effects of WHH1889 on age-related cognitive decline were investigated. WHH1889 was orally administrated (1 × 10^9^ CFU/day) for twelve weeks in aged mice, and their cognitive behaviors, neurochemical factors, cognitive-related gene expressions, neuroinflammation, and serum tryptophan pathway-targeted metabolic profiling, as well as gut microbiome composition were assessed. WHH1889 demonstrated improvement of the cognitive behaviors via the novel object recognition test (NORT), the active shuttle avoidance test (ASAT), the Y-maze test, and the passive avoidance test (PAT). The hippocampal neuronal loss; the declined concentrations of BDNF, 5-HT, and 5-HTP; the decreased gene expressions of neurodegeneration biomarkers; and the increased production of hippocampal inflammatory cytokines in aged mice were restored by WHH1889. In addition, WHH1889 increased the 5-HT/5HTP levels and decreased the serum levels of tryptophan-derived metabolites (e.g., kynurenine, xanthurenic acid, 3-hydroxykynurenine, and 3-hydroxyanthranilic acid). Furthermore, WHH1889 was revealed to shape the gut microbiota community by reversing the relative abundances of Bacteroidota and Firmicutes. The present findings suggest that *L. helveticus* WHH1889 exerted cognitive improving effects on aged mice, which was associated with the modulation of 5-HT and 5-HTP metabolism and gut microbial composition. The supplementation of WHH1889 may therefore be a promising therapeutic agent for age-related cognitive deficits.

## 1. Introduction

The global population is aging; 1 in 6 people in the world will be over 65 by 2050, according to the World Population Prospects (2022 Revision, http://population.un.org/wpp/, accessed on 23 June 2023). Physiological and functional decline, including cognitive decline, are closely linked to aging. Cognitive decline can affect elderly people’s quality of life, which leads to the exhibition of deficits in multiple aspects, such as attention, spatial/non-spatial learning, and memory [1]. The hippocampus has a crucial role in influencing cognitive functions, such as learning and memory, and it is notably vulnerable to the process of aging [2]. Alterations in both the structure and functionality of the hippocampus are linked to the decline in cognitive abilities [1]. Age-related cognitive decline is frequently linked with decreased concentrations of neurotrophic factors, such as brain-derived neurotrophic factor (BDNF), and reduced levels of monoamine neurotransmitters, such as 5-hydroxytryptamine (5-HT), alongside elevated concentrations of inflammatory cytokines, such as interleukin-1β (IL-1β), IL-6, and tumor necrosis factor (TNF)-α within the hippocampus [3,4,5]. Although the complex mechanisms underlying the development of age-related cognitive decline are still unclear, safe and effective treatments to improve cognitive deficits in aging are crucial.

The interaction known as the gut microbiota–brain axis (GBA) involves a bidirectional link between the gut microbiota and the central nervous system (CNS), which can influence both brain and gut functioning [6]; however, the disturbance of the GBA, particularly the alterations in the gut microbial community, affect the microbial-derived cytotoxic metabolite production, resulting in cognitive impairment and neuroinflammation [7]. Recent findings indicate that age-related gut microbial changes can initiate brain aging and age-related cognitive deficits [8]; hence, maintaining a well-balanced gut microbiota is crucial in promoting brain well-being and mitigating cognitive decline associated with aging.

Probiotics, particularly the bacterial strains from the *Lactobacillus* and *Bifidobacterium* genera, are widely recognized for their ability to provide positive impacts on gut health. Certain probiotic strains defined as psychobiotics can shape the various functions in the CNS, such as neuroinflammation, neurotransmission, and neurochemistry through the GBA route [9,10]. Previous studies have revealed that probiotic strains, such as *Lactobacillus helveticus* NS8, *Lacticaseibacillus casei* LC122, *Lacticaseibacillus paracasei* PS23, *Limosilactobacillus fermentum* JDFM216, *Lactiplantibacillus plantarum* NK151 and TWK10, and *Bifidobacterium longum* BL986 and NK173, can improve cognitive impairments in rodents [4,11,12,13,14,15]. These studies indicate that certain psychobiotics may have the potential to mitigate cognitive impairments associated with aging.

*L. helveticus* is recognized as one of the most common probiotics with health-promoting effects [16]. In our recent study, out of twelve *L. helveticus* strains, the *L. helveticus* WHH1889 strain was demonstrated to possess the capability to enhance the production of the precursor to 5-HT, known as 5-hydroxytryptophan (5-HTP), within the colon, exhibiting potential psychobiotic properties, as evidenced by its abilities to modulate the gut microbiota composition and 5-HT/5-HTP metabolism [17]; however, the modulatory role of WHH1889 in cognitive function was not known. A previous study has successfully evaluated the cognitive abilities of *Lactobacillus* and *Bifidobacterium* strains in an aged mouse model using 10-month-old mice for 12 weeks, which can mimic the physiological status of aged mice [11]; therefore, the objective of the current study was to investigate the possible advantages of WHH1889 in alleviating the cognitive decline associated with aging. The changes in cognitive behaviors, the number of hippocampal neurons, neurochemical factors, cognitive-related gene expressions, neuroinflammation, and gut microbiome composition in 10-month-old mice after 12 weeks of WHH1889 administration were evaluated.

## 2. Materials and Methods

### 2.1. Preparation of L. helveticus WHH1889

Preparations of WHH1889 were carried out as per our previous publication [17]. Briefly, WHH1889 was introduced into MRS broth (2% *v*/*v*) and cultivated under anaerobic conditions at 37 °C for 16 h. Subsequent to centrifugation (5000× *g*, 15 min, 25 °C), the culture of WHH1889 was collected, and the sediment was reconstituted in fresh MRS broth consisting of glycerol (30%) to obtain a concentration of 1 × 10^11^ CFU/mL. After being aliquoted in freezer tubes, the WHH1889 was restored at −80 °C immediately. Prior to application, the WHH1889 portion was thawed at 37 °C and centrifugated at 5000× *g* for 15 min to separate the supernatant. The concentration of WHH1889 was then fine-tuned to 5 × 10^9^ CFU/mL using sterile saline. The WHH1889 was stored at the China General Microbiological Culture Collection Centre for preservation (preservation number: CGMCC 21832).

### 2.2. Experiment Design and Sampling

**Figure 1:**
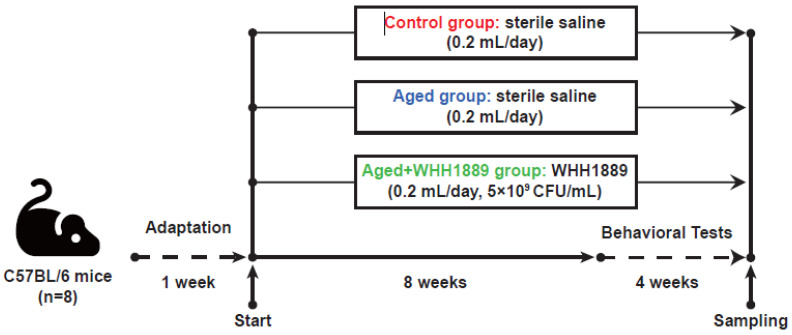
The timeline of the experiment in mice. All mice were acclimatized for one week, and then mice were administered with WHH1889 or saline for a total period of 12 weeks (*n* = 8). Mice were subjected to behavioral tests during the last four weeks of the experiment.

A total of 24 male C57BL/6 mice were acquired from the SLAC Animal Laboratory (Shanghai, China), comprising 8 six-week-old and 16 forty-week-old individuals. The mice were placed in a controlled setting. Specifically, the mice were kept under regulated temperature (26 ± 0.2 °C), humidity (55 ± 10%), and a 12/12 h light/dark cycle (light exposure schedule: 8:30 to 20:30). All the mice were fed with standard rodent chow (p1101f-25, Slaccas, Shanghai, China). The standard chow and water were freely available to the mice at all times. All the animal-related procedures, including care and handling, were conducted in accordance with the Ethics Code Permit WHH2022005 approved by the Animal Care and Use Committee of Hangzhou Wahaha Group Co., Ltd., Hangzhou, China. Following one week of adaptation, the mice were categorized into three groups with 8 replicates: the control group, comprising six-week-old mice; the aged group, consisting of 40-week-old mice; and the aged + WHH1889 group, comprising 40-week-old mice. The sample size was decided in advance in accordance with a previous study [18]. The mice in the aged + WHH1889 group were administered an oral suspension of WHH1889 at a daily dosage of 1 × 10^9^ CFU. The dosage of WHH1889 used in this study was consistent with previous studies conducted on mice [11,17]. Mice in the control and aged groups were given 0.2 mL of sterile saline, which was equal to the amount of WHH1889 given by gavage. All the cognitive behavioral tests were conducted from weeks 9 to 12 (Figure 1).

The mice were euthanized by decapitation 12 weeks after all the cognitive behavioral tests were completed. Blood samples were collected and subjected to centrifugation (3000× *g*, 25 min) to obtain samples of serum. Samples of fresh feces, the hippocampus, and the proximal colon were also collected and kept at −70 °C for later investigations.

### 2.3. Novel Object Recognition Test

The novel object recognition test (NORT) was selected to assess the capabilities of non-spatial learning and memory in mice related to the hippocampus, employing a method previously outlined [11], with some modifications. In brief, the NORT involved placing mice in a hard plastic case (50 cm in length, 50 cm in height, 50 cm in width) with two identical blue wooden cylinders (4 cm in diameter, 12 cm in height) for 10 min per day over four consecutive days. On the fifth day, one cylinder was replaced with a red wooden triangular prism (4 cm in diameter, 12 cm in height) for a 5 min memory test. During the test, the JLBehv-LAM tracking platform (Shanghai Jiliang Software Technology Co., Ltd., Shanghai, China) was applied to record the time that mice spent exploring each object. The recognition index (RI) was computed by dividing the time spent exploring the novel object by the total exploration time. On the other hand, the discrimination index (DI) was determined by dividing the difference in exploration times between the novel and old objects by the total exploration time [11].

### 2.4. Active Shuttle Avoidance Test

The active shuttle avoidance test (ASAT) was performed to assess cognitive ability, as previously described [11]. A shuttle box (30 cm × 20 cm × 40 cm) containing an electric shock grid on the floor and a buzzer with a light source on the top were utilized (JLBehv-STG-4, Jiliang Instruments, Shanghai, China). Over four successive days, each mouse received 20 trials per day of the conditional stimulus (light and sound) and unconditional stimulus (0.2 mA electric shock). The number of active avoidance responses was monitored using the video-tracking platform (JLBehv-STG, Shanghai Jiliang Software Technology, Shanghai, China).

### 2.5. Y-Maze Test

The Y-maze test, employed for the evaluation of exploratory and spatial memory capabilities, was executed using a JLBehv-YM-1 three-arm horizontal maze (40 cm in length, 4 cm in width, and 12 cm in height), following the methodology previously described [19]. The mice were positioned within a single arm of the Y-maze, and their entries into different arms were monitored for a duration of 8 min utilizing the tracking system (JLBehv-YM, Shanghai Jiliang Software Technology). During this assessment, a valid alternation was defined as consecutive entries into all three arms [20]. The calculation for spontaneous alternation (%) was conducted as follows: the count of valid alternations divided by (total arm entries − 2), then multiplied by 100 [20].

### 2.6. Passive Avoidance Test

To evaluate cognitive function, the step-through passive avoidance test (PAT) was carried out using a shuttle box (30 cm in length, 20 cm in width, 40 cm in height) with one bright and one dark compartment (JLBehv-PAG, Shanghai Jiliang Software Technology). According to a previous method [21], initially, every mouse was introduced into the light compartment, and upon complete entry into the dark compartment, they received a 0.2 mA electric shock. The retention test was administered after 24, 48, and 72 h. The JLBehv-PAG tracking system (Shanghai Jiliang Software Technology) was employed to record the latency time, indicating the duration the mouse spent in the light compartment.

### 2.7. Levels of Neurochemical Factors and Cytokines Determination

The hippocampal samples were homogenized at 4 °C by combining them with sterile saline (1:10, *w*/*v*). The supernatants from the samples were collected following centrifugation (5000 rpm, 15 min, 4 °C). Commercial ELISA kits were employed to assess the concentrations of mature BDNF (mBDNF), 5-HT, TNF-α, IL-1β, and IL-6, as per the manufacturer’s protocols (Nanjing Jiancheng Biotech Co., Nanjing, China). The information regarding the precision and accuracy of the ELISA tests can be found in Table S1.

### 2.8. Levels of 5-HTP Evaluation

The concentrations of 5-HTP in the hippocampus and colon were investigated by a documented high-performance liquid chromatography (HPLC) approach, as described previously [22]. The hippocampal and colonic samples were homogenized using a solution of 5% perchloric acid in a ratio of 1 part sample to 10 parts acid (*w*/*v*). Following homogenization, the samples were subjected to centrifugation at 13,000× *g* for 15 min. The resulting supernatant was carefully sampled, then filtered using a 0.22-µm sterile filter, and subsequently kept for further analysis. The levels of 5-HTP in the samples were then analyzed using the Agilent 1220 Infinity LC system (Agilent Technologies, Santa Clara, CA, USA), which was coupled to a fluorescence detector and an analytical C_18_ column (Agilent Technologies).

### 2.9. Hippocampal Neuronal Nissl Staining Analysis

The hippocampal neuronal Nissl staining analysis was perform according to a previous method [23].The number of neuronal cells in the hippocampal key regions, including the cornu ammonis 1 (CA1), CA3, and dentate gyrus (DG), was scored using Image-Pro Plus software (ver. 6.0, Media Cybernetics Inc., Silver Springs, MD, USA).

### 2.10. RNA Extraction and Quantitative RT-PCR

The total RNA from the hippocampal and colonic samples was isolated by the Biozol reagent in accordance with the manufacturer’s instructions (Biomiga, San Diego, CA, USA). The total RNA (approximately 1 µg) from the samples was then transcribed into first-strand cDNA in accordance with the manufacturer’s protocols using a PrimeScript transcription kit (Takara Bio, Shiga, Japan). The primer sequences are shown in Table 1. A quantitative RT-PCR was conducted in triplicate using the SYBR^®^ Premix Ex Taq II kit (Takara Bio, Shiga, Japan) on an ABI Q5 RT-PCR thermocycler system (PE Biosystems, Foster City, CA, USA). The relative expressions were determined utilizing the 2^−ΔΔCycle threshold^ method, with the *Gapdh* transcript serving as the housekeeping gene [24].

### 2.11. Serum Tryptophan Metabolism—Targeted Metabolomic Analysis

The mice serum samples were combined with a precooled extract solution consisting of methanol and acetonitrile (1:4 *v*/*v*), with formic acid (0.1%) and a mixture of internal standards, labeled with isotopes, encompassing a total of 31 metabolites derived from tryptophan (Table S2). Following centrifugation (12,000× *g*, 25 min, 4 °C), approximately 400 μL of supernatant was collected from the samples, evaporated with nitrogen, and redissolved in 100 μL of formic acid (0.1%). The resulting supernatant was acquired via centrifugation (12,000× *g*, 15 min, 4 °C) and, subsequently, underwent analysis using ultra-high-pressure liquid chromatography–mass spectrometry (UHPLC-MS/MS). The separation through UHPLC was conducted using an EXIONLC System (AB Sciex, Foster City, CA, USA), featuring an ACQUITY UPLC HSS T3 column (100 × 2.1 mm, 1.8 μm; Waters, Milford, MA, USA). During the development of the assay, the SCIEX 6500 QTRAP+ triple quadrupole MS (AB Sciex) was employed, coupled with an IonDrive Turbo V electrospray ionization (ESI) interface. Data acquisition and a quantitative analysis were conducted by the SCIEX Analyst Work Station Software (version 1.6.3) and Sciex MultiQuant software (version 3.0.3). A tryptophan metabolism-targeted metabolomic analysis was facilitated with the support of Shanghai Biotree Biotech Co., Ltd. The final concentration (nmol/L) of each metabolite was calculated based on the calculated concentration (nmol/L), the dilution factor (Dil), the volume (μL), and the sample experiment concentration factor using the method previously reported [25]. The results were then normalized using Z-values. In brief, for each tryptophan metabolite, the means with the standard deviation (SD) across all the samples within each group were estimated, and then the Z-value for each concentration value was calculated using the following formula:

Z = (X − mean)/SD

X is the original concentration value.

### 2.12. Gut Microbiome Composition Analysis

To examine the gut microbiome composition in the mice, 16S high-throughput sequencing was employed. The QIAamp DNA Stool Kit was employed to isolate the total genomic DNA from the fecal samples, according to a standard procedure (QIAGEN, Valencia, CA, USA). The 16S rRNA genes of bacteria were amplified using primers with barcodes (27F: 5′-AGRGTTYGATYMTGGCTCAG-3′ and 1492R: 5′-RGYTACCTTGTTACGACTT-3′), which were then purified by the AxyPrep DNA Gel Extraction Kit, following the standard instructions (Axygen Biosciences, Corning, NY, USA). After quantification with QuantiFluorTM-ST (Promega, Madison, WI, USA), the amplicon pools underwent DNA library construction. The SMRTbellTM Template Prep kit 1.0 reagents (Pacific Biosciences, Menlo Park, CA, USA) were utilized in the preparation of the DNA libraries, which were subsequently sequenced using the PacBio Sequel II platform (LC-Bio Technology Co., Ltd., Hangzhou, China). SMRT Link software (version 6.0) was employed to generate circular consensus sequence (CCS) reads from the raw data, which were then filtered for quality using Cutadapt (version 1.9). The resulting CCS reads, with lengths ranging from 1200 to 1650 bp, were retained, and the chimeric sequences were removed using the DADA2 pipeline in QIIME 2 (version 2022.2). Clean amplicon sequencing variants (ASVs) sequences were obtained and taxonomy annotation of the ASVs was performed using the SILVA database (release 138). The sequences produced throughout this research have been submitted to the NCBI Sequence Read Archive (SRA) database (accession number PRJNA937416). The analysis of the 16S rRNA data was carried out using QIIME 2, while visualization was accomplished by the R package (ggplot2, version 3.1.1, https://cran.r-project.org/ggplot2, accessed on 14 October 2022), according to the standard pipeline.

### 2.13. Statistical Analysis

The data underwent analysis on the R software platform (version 4.0.3) and were assessed for normal distribution by the Shapiro–Wilk test. Statistical examinations were carried out employing the Student’s *t*-test or the Mann–Whitney *U* Test for comparisons between the two groups, while the one-way analysis of variance (ANOVA) or Kruskal–Wallis test was applied for comparisons among all the groups, based on the outcomes of the distribution test. The analysis results were shown as medians alongside 95% confidence intervals (Cis). The significance was determined when the *p* values < 0.05. Following the correction of the *p* values for a false discovery rate (FDR) [26], the reported q values were considered significant when below 0.05.

## 3. Results

### 3.1. Effects of WHH1889 on Cognitive Behaviors in Aged Mice

**Figure 2:**
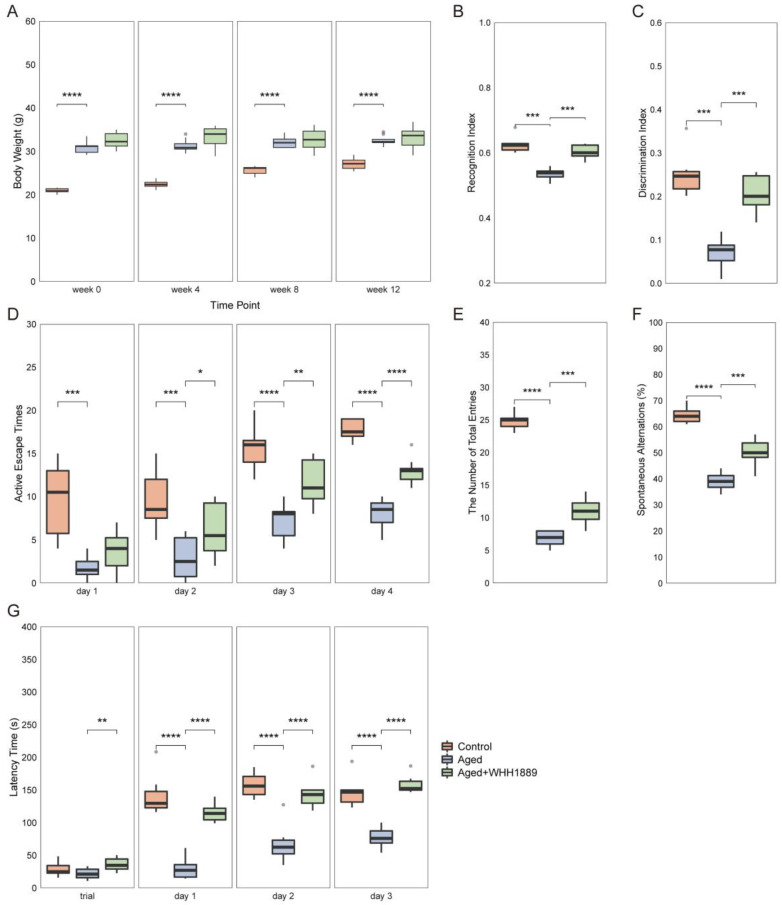
The changes in the cognitive behaviors in aged mice after WHH1889 administration. (**A**) Body weight. (**B**) The recognition index and (**C**) discrimination index in the novel object recognition test (NORT). (**D**) The active escape times (frequency) in the active shuttle avoidance test (ASAT). (**E**) The number of total arm entries and (**F**) spontaneous alternations in the Y-maze test. (**G**) The latency time in passive avoidance test (PAT). Results are expressed as medians ± 95% CI, *n* = 8. * *p* < 0.05, ** *p* < 0.01, *** *p* < 0.001, **** *p* < 0.0001.

Throughout the experiment, the aged mice showed a higher body weight compared with the young mice (Figure 2A, *p* < 0.0001), whereas WHH1889 did not demonstrate significant modulatory effects on the body weight of the aged mice (Figure 2A, *p* > 0.05). Cognitive behavioral tests were conducted to investigate the learning ability and memory function of aged mice treated with WHH1889. In the NORT, compared with the control group, the aged mice exhibited significantly lower RI and DI (Figure 2B,C, *p* < 0.001), and both RI and DI were increased after WHH1889 administration in the aged mice (Figure 2B,C, *p* < 0.001). During the ASAT, the active escape times were significantly reduced in the aged mice from day 1 to day 4 (Figure 2D, *p* < 0.001), and significantly increased on day 2 (*p* < 0.05), day 3 (*p* < 0.01) and day 4 (*p* < 0.0001) with WHH1889 treatment (Figure 2D). In the Y-maze test, compared with the control group, mice in the aged group exhibited a significant decrease in both the number of total arm entries and spontaneous alternations (%) (Figure 2E,F, *p* < 0.0001), and oral administration of WHH1889 significantly increased the number of arm entries and alternations in the aged mice (Figure 2E,F, *p* < 0.001). During the PAT, the latency time was significantly reduced in the aged mice from day 1 to day 3 (Figure 2G, *p* < 0.0001), and restored significantly by WHH1889 treatment from day 1 to day 3 (Figure 2G, *p* < 0.0001).

### 3.2. Effects of WHH1889 on the Number of Hippocampal Neurons in Aged Mice

The alterations in the number of hippocampal neuronal cells in the aged mice after WHH1889 treatment were investigated. The key hippocampal regions responsible for cognitive function, including CA1, CA3, and DG, were selected for neuronal cell scoring (Figure S1A). Compared to the young mice, the numbers of neurons in the hippocampal CA1, CA3, and DG areas were significantly lower in the aged mice (Figure S1B–D, *p* < 0.0001). In addition, following the WHH1889 administration, the aged mice presented a significantly higher number of neuronal cells in the hippocampal CA1, CA3, and DG areas (Figure S1B–D, *p* < 0.001). These results indicate that WHH1889 played a beneficial role in mitigating hippocampal neuronal loss in the aged mice.

### 3.3. Effects of WHH1889 on Neurochemical Factors, Gene Expressions, and Neuroinflammation in Aged Mice

**Figure 3:**
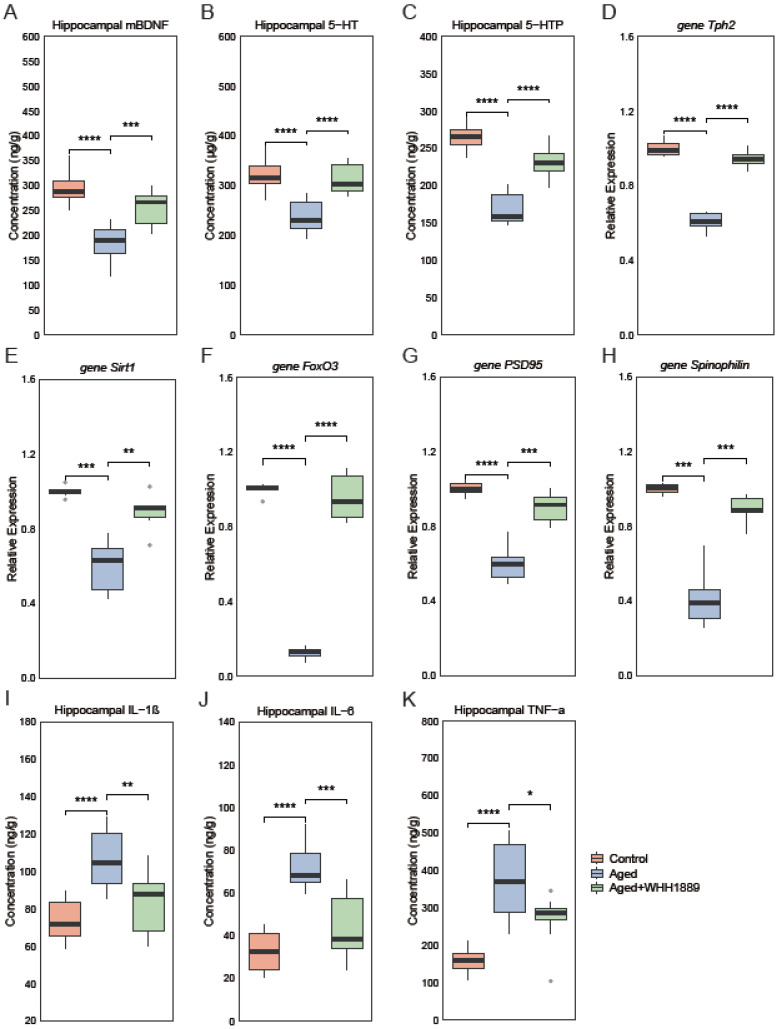
The alterations in the levels of neurochemical parameters, gene expressions, and neuroinflammation in aged mice after WHH1889 administration. (**A**) Levels of mature BDNF (mBDNF) in the hippocampus. (**B**) Levels of 5-HT in the hippocampus. (**C**) Levels of 5-HTP in the hippocampus. (**D**) The gene expressions of *Tph2* in the hippocampus. The gene expressions of *Sirt1* (**E**), *FoxO3* (**F**), *PSD95* (**G**), and *Spinophilin* (**H**) in the hippocampus. The levels of IL-1β (**I**), IL-6 (**J**), and TNF-α (**K**) in the hippocampus. Results are expressed as medians ± 95% CI, *n* = 8. * *p* < 0.05, ** *p* < 0.01, *** *p* < 0.001, **** *p* < 0.0001. Regarding the concentrations of mBDNF, 5-HT, and 5-HTP in the hippocampus, the units “ng/g” and “µg/g” indicate nanograms or micrograms per gram of fresh tissue, respectively.

The changes in cognitive-related neurochemical factors in the hippocampus were examined. Compared with the control group, mice in the aged group exhibited significantly lower hippocampal concentrations of mBDNF, 5-HT, and 5-HTP (Figure 3A–C, *p* < 0.0001). In contrast, the administration of WHH1889 significantly increased the levels of mBDNF (Figure 3A, *p* < 0.001), 5-HT (Figure 3B, *p* < 0.0001), and 5-HTP (Figure 3C, *p* < 0.0001) in the hippocampuses of the aged mice. Tryptophan hydroxylase (Tph) has two subtypes, namely, Tph1 and Tph2. Tph1 is dominantly expressed in the gut, while Tph2 is primarily present in the brain and is responsible for the synthesis of 5-HT [27]. The hippocampal *Tph2* gene expression was significantly down-regulated in the aged mice (Figure 3D, *p* < 0.0001) and restored after administration of WHH1889 (Figure 3D, *p* < 0.0001).

The changes in gene expressions of the biomarkers associated with neurodegeneration were analyzed in the hippocampus. The gene expressions of *Sirt1* (Figure 3E, *p* < 0.001), *FoxO3* (Figure 3F, *p* < 0.0001), *PSD95* (Figure 3G, *p* < 0.0001), and *Spinophilin* (Figure 3H, *p* < 0.001) were significantly reduced in the aged mice and significantly reversed by the WHH1889 treatment (Figure 3E,H, *p* < 0.01).

The changes in hippocampal neuroinflammation were also examined. The aged mice exhibited higher productions of hippocampal cytokines (Il-1β, IL-6, and TNF-α) than the young mice (Figure 3I–K, *p* < 0.0001). Conversely, the administration of WHH1889 significantly decreased the levels of these cytokines in the aged mice (Figure 3I–K, *p* < 0.05).

### 3.4. Effects of WHH1889 on Serum Tryptophan Metabolism in Aged Mice

**Figure 4:**
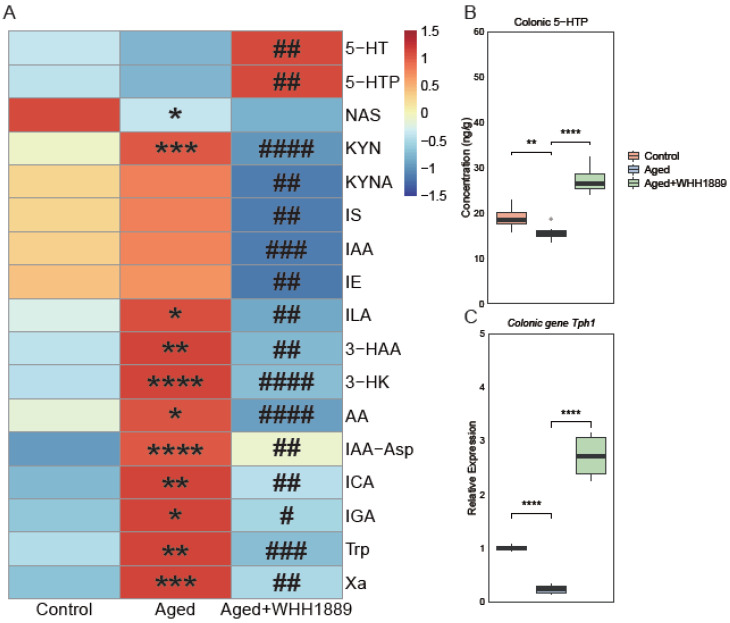
(**A**) The significantly changed metabolites in the serum tryptophan metabolism revealed by the targeted metabolomics analysis (*n* = 8). The concentrations of tryptophan metabolites were normalized using Z values; the red cell indicates a higher level, and the blue cell indicates a lower level. * *p* < 0.05, ** *p* < 0.01, *** *p* < 0.001, **** *p* < 0.0001 vs. control group; # *p* < 0.05, ## *p* < 0.01, ### *p* < 0.001, #### *p* < 0.0001 vs. aged group. (**B**) Levels of 5-HTP in the colon. (**C**) The gene expressions of *Tph1* in the colon. Results are expressed as medians ± 95% CI, *n* = 8. * *p* < 0.05, ** *p* < 0.01, *** *p* < 0.001, **** *p* < 0.0001. Regarding the concentration of 5-HTP in the colon, the unit “ng/g” indicates nanograms per gram of fresh tissue.

The changes in the serum tryptophan metabolism were revealed by a tryptophan metabolism-targeted metabolomic analysis. Seventeen tryptophan metabolites were significantly changed among the groups (Figure 4A, Table S3). Compared to the control group, the aged mice exhibited significantly lower concentrations of N-acetyl-5-HT (NAS, *p* < 0.05) and higher levels of various metabolites, including kynurenine (KYN, *p* < 0.001), indolelactic acid (ILA, *p* < 0.05), 3-hydroxyanthranilic acid (3-HAA, *p* < 0.01), 3-hydroxykynurenine (3-HK, *p* < 0.0001), anthranilic acid (AA, *p* < 0.05), indole-3-acetyl-aspartate (IAA-Asp, *p* < 0.0001), indole-3-carboxaldehyde (ICA, *p* < 0.01), 3-indoleglyoxylic acid (IGA, *p* < 0.05), tryptophan (*p* < 0.01), and xanthurenic acid (Xa, *p* < 0.001). The oral administration of WHH1889 significantly increased the serum levels of 5-HT (*p* < 0.01) and 5-HTP (*p* < 0.01), and decreased the levels of several metabolites, including KYN (*p* < 0.0001), kynurenic acid (KYNA, *p* < 0.01), indoxyl sulfate (IS, *p* < 0.01), indole-3-acetic acid (IAA, *p* < 0.001), indole ethanol (IE, *p* < 0.01), ILA (*p* < 0.01), 3-HAA (*p* < 0.01), 3-HK (*p* < 0.0001), AA (*p* < 0.0001), IAA-Asp (*p* < 0.01), ICA (*p* < 0.01), IGA (*p* < 0.05), tryptophan (*p* < 0.001), and Xa (*p* < 0.01) in the aged mice.

### 3.5. Effects of WHH1889 on the Level of 5-HTP and Gene Expression of Tph1 in the Colon of Aged Mice

Given that WHH1889 can promote 5-HTP secretion in the colon, the level of 5-HTP and the gene expression of *Tph1* in the proximal colon were assessed. Tph1 is the key synthetase expressed in the gut for the biosynthesis of 5-HT [27]. Compared with the control group, the aged mice showed lower concentrations of 5-HTP (Figure 4B, *p* < 0.01) and reduced *Tph1* gene expression (Figure 4C, *p* < 0.0001) in the colon; however, the oral administration of WHH1889 significantly increased both the production of 5-HTP and the gene expression of *Tph1* in the colons of the aged mice (Figure 4B,C, *p* < 0.0001).

### 3.6. Impacts of WHH1889 on the Gut Microbial Community in Aged Mice

**Figure 5:**
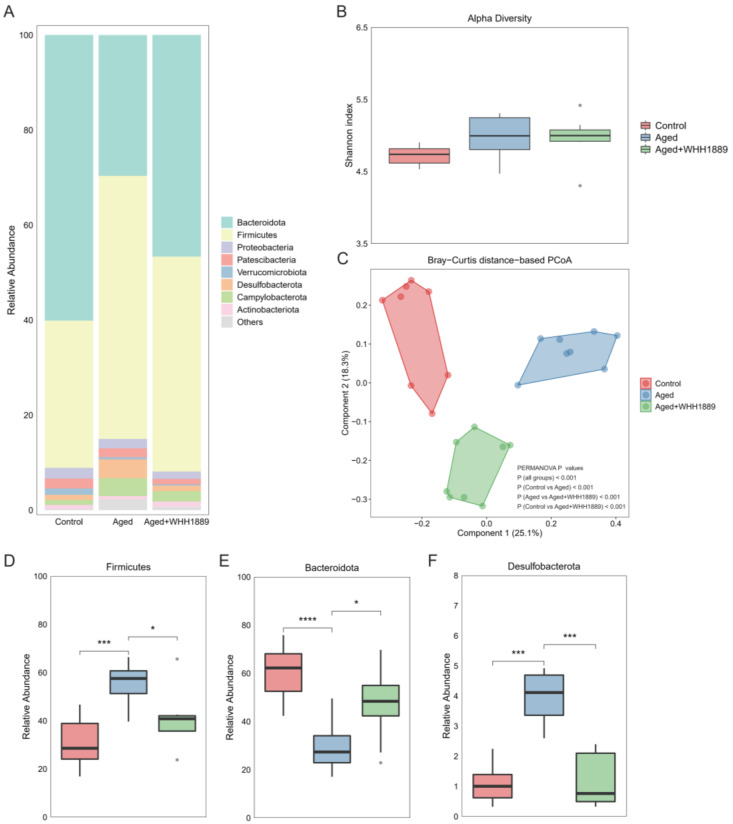
The alterations in the gut microbiome composition in aged mice after WHH1889 administration. (**A**) The composition of gut microbiome at the phylum level. (**B**) The Shannon indices of the gut microbial community. (**C**) The gut microbial β diversity was revealed by the Bray–Curtis distance-based PCoA. PERMANOVA analysis was performed at the feature level. *p* < 0.05 indicates the significant difference between/within groups. The significant changes in the abundance of phyla Firmicutes (**D**), Bacteroidota (**E**), and Desulfobacterota (**F**). Results are expressed as medians ± 95% CI, *n* = 8. * *p* < 0.05, *** *p* < 0.001, **** *p* < 0.0001.

Alterations in the gut microbial structure of the aged mice following treatment with WHH1889 were examined (Figure 5A). The gut microbial α diversity did not exhibit significant changes among the groups (Figure 5B, *p* > 0.05), while notable differences in the gut microbial β diversity were observed, as evidenced by the distinct separations among the groups in the Bray–Curtis distance-based PCoA (Figure 5C, PERMANOVA *p* < 0.001).

**Figure 6:**
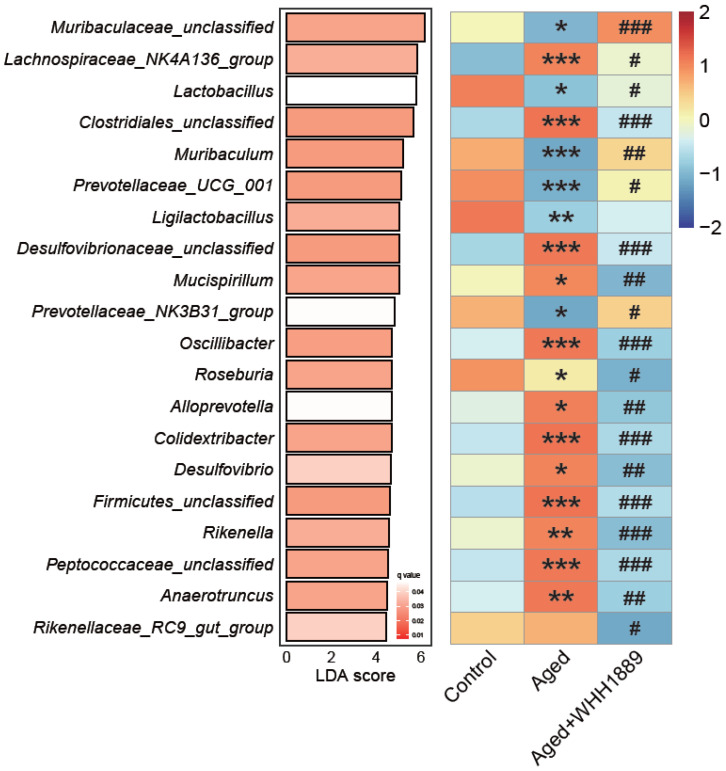
Top 20 significantly changed gut microbial genera in aged mice after WHH1889 administration. The differential alterations at the genus level were revealed using LefSe analysis with an LDA score > 3.0 and q value < 0.05. Results were normalized into Z values; red cells suggest higher abundance, and blue cells suggest lower abundance. * *p* < 0.05, ** *p* < 0.01, *** *p* < 0.001 vs. control group; # *p* < 0.05, ## *p* < 0.01, ### *p* < 0.001 vs. aged group.

The results reveal that the aged mice had higher levels of Firmicutes (Figure 5D, *p* < 0.001) and Desulfobacterota (Figure 5F, *p* < 0.001), and lower levels of Bacteroidota (Figure 5E, *p* < 0.0001) when compared to the young mice; however, the oral administration of WHH1889 significantly reverted the alterations in the abundance of these dominant phyla in the aged mice (Figure 5D–F, *p* < 0.05). The linear discriminant analysis effect size (LefSe) with an LDA score > 3.0 and q value < 0.05 identified the top 20 significantly changed genera at the genus level (Figure 6, Table S4). In the aged mice, compared with the young mice, the reduced abundance of unclassified *Muribaculaceae* (*p* < 0.05), *Lactobacillus* (*p* < 0.05), *Muribaculum* (*p* < 0.001), *Prevotellaceae_UCG_001* (*p* < 0.001), and *Prevotellaceae_NK3B31_group* (*p* < 0.05), and the increased abundance of *Lachnospiraceae_NK4A136_group* (*p* < 0.001), unclassified *Clostridiales* (*p* < 0.001), unclassified *Desulfovibrionaceae* (*p* < 0.001), *Mucispirillum* (*p* < 0.05), *Oscillibacter* (*p* < 0.001), *Roseburia* (*p* < 0.05), *Alloprevotella* (*p* < 0.05), *Colidextribacter* (*p* < 0.001), *Desulfovibrio* (*p* < 0.05), unclassified *Firmicutes* (*p* < 0.001), *Rikenella* (*p* < 0.01), unclassified *Peptococcaceae* (*p* < 0.001), and *Anaerotruncus* (*p* < 0.01) were observed. Nevertheless, after treatment with WHH1889, the shifts in the abundance of these genera were reverted (Figure 6, *p* < 0.05).

## 4. Discussion

With the global aging population on the rise, it is important to develop novel therapeutic approaches for cognitive decline. Our recent study has shown that *L. helveticus* WHH1889 possesses antidepressant-like effects in depressed mice [17], indicating its potential as a psychobiotic agent. Building on this, this research has demonstrated that the administration of WHH1889 can mitigate age-related cognitive decline in mice. Specifically, after 12 weeks of treatment with WHH1889, the impaired cognitive behaviors, hippocampal neuronal loss, low concentrations of hippocampal 5-HT, 5-HTP, and mBDNF, high levels of neuroinflammation, disturbed serum tryptophan metabolism, and altered gut microbiome composition in the aged mice were improved. The current findings indicate that WHH1889 may offer a potential as a therapeutic agent for treating cognitive decline associated with aging.

Aging is a natural process that often results in cognitive decline, which can cause deficits in learning and memory [1]. In line with previous studies [11,28], our study found that aged mice exhibited clear impairments in cognitive function, including low RI and DI scores in the NORT, reduced spontaneous alternation in the Y-maze test, as well as decreased activity levels during the ASAT and shorter latency times in the PAT. The administration of WHH1889 was effective at mitigating these cognitive deficits in the aged mice, highlighting its potential as a cognitive-enhancing supplement. Our findings are in line with earlier studies revealing that strains of *L. helveticus*, such as *L. helveticus* NS8, have abilities to attenuate cognitive impairments in mice [14], pointing to the potential cognitive improving properties among the *L. helveticus* strains.

The hippocampus is one of the pivotal brain regions and is involved in functional processes of cognition [1,2]. It is particularly susceptible to age-related decline, as evidenced by the impaired memory and learning ability in aging populations [29]. The present study found decreased gene expressions of several neurodegenerative biomarkers in the hippocampus of aged mice, including *Sirt1* and *FoxO3*, which have been revealed to modulate cognitive decline [30], and reduced expressions of synapse-related genes *PSD95* and *Spinophilin*, which play crucial roles in synaptic plasticity modulation [21,31]. These findings suggest the presence of cognitive deficits in aged mice. Interestingly, these gene expressions were up-regulated after oral administration of WHH1889. Consistent with our findings, the decreased hippocampal gene expressions of these cognitive-related biomarkers, such as *Sirt*, *FoxO3*, and *PSD95*, in aged mice can be normalized by certain probiotic strains [11,32]. The current findings indicate that WHH1889 may improve cognitive behaviors by modulating the neurodegenerative gene expressions in the hippocampus.

BDNF, a member of the neurotrophic factors family, is crucial in promoting neurogenesis, synaptic plasticity, and neuronal survival, which contribute to improved cognitive function [5]. Reduced BDNF levels are strongly linked to age-related cognitive decline in the hippocampus [33]. The current study, in agreement with prior studies [3,11], revealed low levels of hippocampal BDNF in the aged mice. Certain probiotic strains, such as *L. casei* LC122 and *B. longum* BL986, have been observed to enhance learning and memory abilities by elevating the levels of hippocampal BDNF in aged mice [11]. Similarly, the present study showed that WHH1889 administration partially reversed the decline in hippocampal BDNF levels, suggesting that BDNF could be a crucial factor in the beneficial effects of WHH1889 on cognitive decline associated with aging.

Growing evidence has revealed low-grade neuroinflammation with aging [34]. In our investigation, elevated concentrations of cytokines were observed in the hippocampus of aged mice. Probiotics are effective in reducing inflammation in both the gut and the CNS through the GBA pathway [6,9]. Earlier studies have also revealed that a certain probiotic strain supplementation can mitigate neuroinflammation [11,13]. The oral administration of WHH1889 consistently reduced the hippocampal levels of inflammatory cytokines, pointing to a potential anti-inflammatory role for WHH1889 in aged mice.

The interaction between brain function and gut microbiota involves tryptophan metabolism. As revealed by the tryptophan metabolism-targeted metabolomics, the modulatory role of WHH1889 on tryptophan metabolism is highlighted. As an essential amino acid, tryptophan can be metabolized via three routes, including the KYN metabolism pathway, the 5-HT synthesis pathway, and the microbial metabolism pathway [27]. In this current study, we noted an up-regulation of the KYN metabolism pathway in the aged mice, demonstrated by elevated serum levels of KYN, 3-HAA, 3-HK, and Xa. Nevertheless, excessive activation of the KYN pathway has been linked to impairments in learning and memory, mainly attributed to the neurotoxic consequences of 3-HAA [35]. Notably, the administration of WHH1889 reversed the changes in KYN pathway metabolites, indicating its critical role in modulating tryptophan metabolism in aged mice.

The gut microbiota is known to have a crucial role in shaping gut homeostasis in the host, as well as in CNS function [6]. The gut microbial composition and function undergo profound remodeling with aging [36]. Furthermore, gut dysbiosis has been observed in rodents [4,11,15] and humans [37] with age-related cognitive decline. In the current investigation, evident disparities in the gut microbiome composition between young and aged mice were observed. The two most dominant phyla in the mouse gut microbiota are Bacteroidota and Firmicutes, and the aged mice showed reduced Bacteroidota abundance and elevated Firmicutes abundance. Similar to our findings, this shift pattern has been reported previously [15], while the opposite results have also been revealed [4]. Interestingly, it is worth noting that a previous study has shown that the abundances of Bacteroidota and Firmicutes are unaffected by aging [11]. This lack of consensus may be due to a variety of factors, such as diet, genetic background, antibiotics, and age [38]. In our study, the administration of WHH1889 reversed the altered abundances of Bacteroidota and Firmicutes. This is consistent with similar studies using probiotic strains, including *L. plantarum* TWK10 [15] and *L. paracasei* PS23 [4], to normalize the changes in Bacteroidota and Firmicutes.

The effects of WHH1889 on the dominant genera in the gut were disclosed using LefSe analysis. In particular, the dominant genus *Lactobacillus* is recognized to have beneficial effects on the host’s cognitive function [9]. The decreased *Lactobacillus* abundance in the aged mice can be reversed by the WHH1889 administration, pointing to the possible mechanism that WHH1889 may partly improve cognitive function by promoting gut *Lactobacillus* abundance. The genus *Muribaculum* is predominant in the gut of mammals, which has been reported to be enriched in cognitive disease and negatively linked with cognitive decline in mice [39]. In the present study, the increased abundance of *Muribaculum* was restored in the aged mice by the WHH1889 treatment. Other dominant genera, including *Mucispirillum* [40], *Oscillibacter* [41], *Alloprevotella* [42], and *Desulfovibrio* [43], have also been shown to be associated with cognitive functions in rodents. In our investigation, the diminished abundances of these genera were restored following the oral administration of WHH1889. This indicates their potential involvement in facilitating the cognitive-enhancing effects of WHH1889.

The 5-HT metabolism is crucial for mood and cognition function. 5-HT, one of the monoamine neurotransmitters, is synthesized mainly from tryptophan, with 5-HTP as an intermediate factor [5]. In the hippocampus, native 5-HT can enhance long-term potentiation, while only 5-HTP and tryptophan can cross the brain–blood barrier [44]. Age-related cognitive decline has been closely linked with dysregulation of 5-HT metabolism [4], as confirmed by our recent study, in which we observed low concentrations of hippocampal 5-HT and 5-HTP in aged mice. Our recent study has revealed that WHH1889 can effectively enhance hippocampal 5-HT and 5-HTP productions by stimulating colonic 5-HTP synthesis [17]. Interestingly, the tryptophan-targeted metabolomic analysis showed elevated serum levels of 5-HT and 5-HTP in aged mice following treatment with WHH1889; therefore, this study provides further evidence that WHH1889 may modulate the central 5-HT metabolism through the 5-HTP-dependent pathway. This potential mechanism will be validated further.

Several limitations should be acknowledged in the current study. Firstly, the intricate and reciprocal interaction between the gut microbial community and CNS function via the gut–brain axis (GBA) [6] makes it challenging to conclude that WHH1889 improved the cognitive function in the aged mice solely by shaping the gut microbiome composition. Secondly, although the present research provided evidence supporting the therapeutic promise of WHH1889 for cognitive decline associated with aging, the precise underlying mechanisms remain ambiguous and require further investigation. Thirdly, randomized controlled trials should be performed to validate the findings in animal models.

## 5. Conclusions

**Figure 7:**
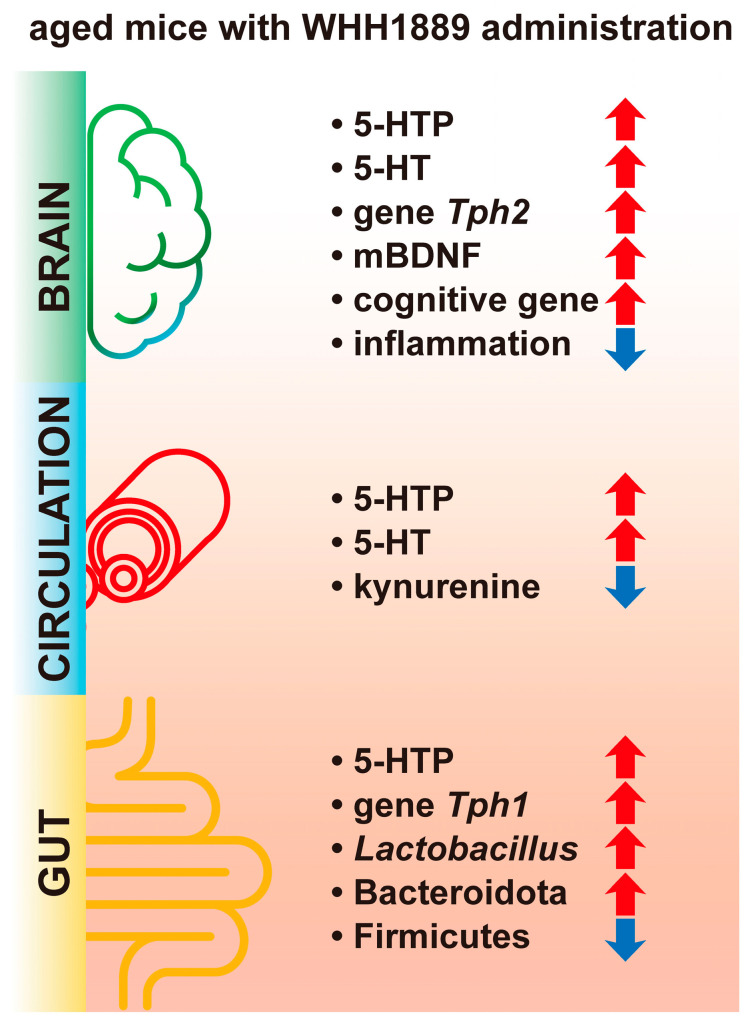
A summary diagram of the cognitive-improving effects of WHH1889 on aged mice. Increased factors are marked with red arrows, and decreased factors are marked with blue arrows.

In summary, our study reveals that WHH1889 improves cognitive behaviors, hippocampal neuronal loss, hippocampal BDNF and 5-HT metabolism abnormalities, neuroinflammation, and serum tryptophan metabolism disturbances in aged mice. The cognitive-improving effects of WHH1889 are associated with the modulation of 5-HT metabolism and the gut microbiome composition (Figure 7); therefore, ingestion of WHH1889, which possesses psychobiotic properties, holds potential as a promising therapeutic approach for age-related cognitive decline.

## Figures and Tables

**Table 1:** Information about primers used in this study.

Primer Name	Primer Sequences	GenBank Accession	Annealing Temperature (°C)
*Sirt1*	F-5′-TCCTTGGAGACTGCGATGTTA-3′ R-5′-GTGGCAACTCTGATAAATGAACC-3′	NM_001159589.2	60
*FoxO3*	F-5′-GGACGACCTGCTGGATAACAT-3′ R-5′-CCTGGATAGTCTGCATGGGTG-3′	NM_001376967.1	60
*PSD95*	F-5′-GGGAGATGGAGTATGAGGAGA-3′ R-5′-TGATAAAGATGGATGGGTCGT-3′	NM_001109752.1	60
*Spinophilin*	F-5′-AGGACTATGACCGACGCAATG-3′ R-5′-TCCAGCTCCACAGGAAACAG-3′	NM_172261.3	60
*Tph2*	F-5′-TGGAGCAGGGTTACTTTCGT-3′ R-5′-AAGCAGGTCGTCTTTGGGT-3′	NM_173391.3	60
*Tph1*	F-5′-AACAAAGACCATTCCTCCGAAAG-3′ R-5′-TGTAACAGGCTCACATGATTCTC-3′	NM_001136084	60
*Gapdh*	F-5′-AACAGCAACTCCCACTCTTCC-3′ R-5′-TGGTCCAGGGTTTCTTACTCC-3′	NM_008084.4	60

## Data Availability

The 16S high-throughput sequencing data are available on the NCBI SRA database under accession number PRJNA937416. The datasets used and/or analyzed during the current study are available from the corresponding author upon reasonable request.

## Supplementary Materials

The following supporting information can be downloaded at: https://www.mdpi.com/article/10.3390/nu15173852/s1, Figure S1: Effects of WHH1889 on the number of hippocampal neurons in aged mice; Table S1: The precision and accuracy of the ELISA assays in this study; Table S2: Information on the standard compounds of tryptophan metabolites; Table S3: The significantly changed tryptophan metabolites; Table S4: The top 20 significantly changed microbial genera revealed by the LefSe method.
