# Supplementary material for: Improvements of Age-Related Cognitive Decline in Mice by Lactobacillus helveticus WHH1889, a Novel Strain with Psychobiotic Properties
Source: Nutrients. 2023 Sep 3;15(17):3852. doi: 10.3390/nu15173852 (PMC10489973; doi:10.3390/nu15173852)
Supplement: Supplementary file 1 [file nutrients-15-03852-s001.zip › nutrients-2564932-supplementary.pdf]

Supplemental Information

Improvements of age-related cognitive decline in mice by *Lactobacillus helveticus* WHH1889, a novel strain with psychobiotic properties

Kan Gao [1,2,3] , Cailing Chen [1,2], Xueqin Ke [1,2], Qiuling Fan [1,2], Haifeng Wang [3,*] , Yanjun Li [1,2,4] and Su Chen [1,2,*]

[1] Research and Development Department, Hangzhou Wahaha Group Co., Ltd., Hangzhou 310018, China; kevingogh911@hotmail.com (K.G.); chencailing@wahaha.com.cn (C.C.); xueqin.ke@wahaha.com.cn (X.K.); qiuling.fan@wahaha.com.cn (Q.F.); lyj@wahaha.com.cn (Y.L.)

[2] Key Laboratory of Food and Biological Engineering of Zhejiang Province, Hangzhou 310018, China

[3] MOE Key Laboratory of Molecular Animal Nutrition, College of Animal Science, Zhejiang University, Hangzhou 310058, China

[4] College of Biosystems Engineering and Food Science, Zhejiang University, Hangzhou 310058, China

***** Correspondence: haifengwang@zju.edu.cn (H.W.); chensu@wahaha.com.cn (S.C.)

SUPPLEMENTARY TABLES

Table S1. The precision and accuracy of the ELISA assays in this study

Items	r2	Precision (%)1	Accuracy (%)2
Hippocampal mBDNF	0.9997	3.23	5.23
Hippocampal 5-HT	0.9996	4.12	5.57
Hippocampal IL-1β	0.9999	2.89	4.22
Hippocampal IL-6	0.9998	3.12	4.65
Hippocampal TNF-α	0.9999	2.98	4.58
Colonic 5-HTP	0.9999	2.78	4.23

[1] The precision (agreement between replicate measurements) of the method, as evaluated by the relative deviation (mean of absolute deviation/mean of replicate measurements × 100%). A value below 5 % indicates the method is highly precise.

[2] The accuracy (the closeness of an experimental value to the true value) of the method, as determined with known amounts of neurochemical factors standards and expressed as the relative errors [(measurement value – true value)/true value × 100%)]. A value below 10 % indicates the method is highly accurate.

Table S2. Information on the standard compounds of tryptophan metabolites

Metabolites	Abbreviation	CAS Number	Formula
3-Hydroxyanthranilic acid	3-HAA	548-93-6	C7H7NO3
3-Hydroxykynurenine	3-HK	2147-61-7	C10H12N2O4
5-Hydroxyindoleacetic acid	5-HIAA	54-16-0	C10H9NO3
Serotonin	5-HT	50-67-9	C10H12N2O
5-Hydroxytryptophol	5-HTOL	154-02-9	C10H11NO2
L-5-Hydroxytryptophan	5-HTP	4350-09-8	C11H12N2O3
5-Methoxy-3-indoleacetic acid	5-Me-IAA	3471-31-6	C11H11NO3
Anthranilic acid	AA	118-92-3	C7H7NO2
Indole acrylic acid	IA	29953-71-7	C11H9NO2
Indole-3-acetic acid	IAA	87-51-4	C10H9NO2
Indole-3-acetamide	IAM	879-37-8	C10H10N2O
Indole-3-carboxaldehyde	ICA	487-89-8	C9H7NO
Indole ethanol/tryptophol	IE	526-55-6	C10H11NO
3-Indoleglyoxylic acid	IGA	1477-49-2	C10H7NO3
Indolelactic acid	ILA	1821-52-9	C11H11NO3
Indican	Indican	487-60-5	C14H17NO6
Indole	Indole	120-72-9	C8H7N
3-Indolepropionic acid	IPA	830-96-6	C11H11NO2
Indoxylsulfate	IS	2642-37-7	C8H7NO4S
Kynurenine	KYN	343-65-7	C10H12N2O3
Kynurenic acid	KYNA	492-27-3	C10H7NO3
Melatonin	Melatonin	73-31-4	C13H16N2O2
Nicotinic acid	NA	59-67-6	C6H5NO2
N-Acetyl-5-hydroxytryptamine	NAS	1210-83-9	C12H14N2O2
Skatole	Skatole	83-34-1	C9H9N
L-Tryptophan	Trp	73-22-3	C11H12N2O2
Tryptamine	Tryptamine	61-54-1	C10H12N2
Xanthurenic acid	Xa	59-00-7	C10H7NO4
Indole-3-acetonitrile	IAN	771-51-7	C10H8N2
Indole-3-acetyl-alanine	IAA-Ala	57105-39-2	C13H14N2O3
Indole-3-acetyl-aspartate	IAA-Asp	2456-73-7	C14H14N2O5

Table S3. The significantly changed tryptophan metabolites

Metabolites (nmol/L)	Groups			*P* value	q value[1]
	Control	Aged	Aged+WHH1889		
5-HT	13827.56±350.25[b]	13509.29±217.08[b]	15056.95±262.81[a]	0.0010	0.0021
5-HTP	183.04±4.78[b]	173.67±8.51[b]	226.45±17.22[a]	0.0100	0.0131
NAS	21.66±1.92[a]	17.22±1.05[b]	16.13±0.77[b]	0.0200	0.0213
KYN	992.81±45.59[b]	1320.60±60.10[a]	789.81±33.81[c]	0.0000	0.0001
KYNA	157.18±13.86[b]	184.53±23.74[a]	97.96±10.83[c]	0.0120	0.0146
IS	21443.57±2454.10[a]	23895.37±1591.34[a]	15685.47±1475.61[b]	0.0170	0.0193
IAA	771.99±86.51[a]	936.45±101.21[a]	404.02±38.18[b]	0.0004	0.0021
IE	1.20±0.22[a]	1.35±0.13[a]	0.60±0.05[b]	0.0050	0.0077
ILA	1980.60±106.38[b]	2489.95±209.42[a]	1781.18±121.58[b]	0.0100	0.0131
3-HAA	20.04±2.25[b]	46.62±10.15[a]	16.73±2.44[b]	0.0050	0.0077
3-HK	143.48±10.23[b]	315.64±31.72[a]	125.50±17.17[b]	0.0000	0.0001
AA	51.70±5.05[b]	74.19±5.81[a]	41.29±4.67[b]	0.0010	0.0021
IAA-Asp	0.40±0.16[b]	1.97±0.31[a]	0.79±0.29[b]	0.0010	0.0021
ICA	269.00±22.60[b]	405.58±29.46[a]	288.08±29.37[b]	0.0040	0.0076
IGA	52.60±5.40[b]	76.63±8.44[a]	53.96±5.30[b]	0.0280	0.0280
Trp	112092.82±3681.80[b]	140577.15±6487.28[a]	108475.90±6200.60[b]	0.0010	0.0021
Xa	314.34±14.92[b]	436.01±18.37[a]	323.93±30.60[b]	0.0010	0.0021

[1] q values were calculated based on the *P* values with FDR correction.

Table S4. The top 20 significantly changed microbial genera revealed by the LefSe method[1]

Top 20 Significantly Changed Genera	Groups			LDA score	*P* value	q value[2]
	Control	Aged	Aged+WHH1889			
unclassified Muribaculaceae	33.70±4.35[b]	20.93±2.92[c]	50.52±3.47[a]	6.17	0.0013	0.0024
Lachnospiraceae_NK4A136_group	5.57±1.32[c]	19.32±2.15[a]	9.27±2.98[b]	5.84	0.0026	0.0035
Lactobacillus	15.04±7.26[a]	2.62±1.04[c]	4.82±0.92[b]	5.79	0.0235	0.0235
unclassified Clostridiales	1.92±0.17[b]	10.90±2.07[a]	2.22±0.51[b]	5.65	0.0005	0.0020
Muribaculum	4.62±0.54[a]	1.49±0.18[b]	3.80±0.43[a]	5.19	0.0005	0.0020
Prevotellaceae_UCG_001	2.96±0.48[a]	0.39±0.11[c]	1.20±0.31[b]	5.11	0.0005	0.0020
Ligilactobacillus	3.06±0.53[a]	0.86±0.22[b]	1.13±0.22[b]	5.04	0.0023	0.0033
unclassified Desulfovibrionaceae	0.59±0.18[b]	2.70±0.30[a]	0.71±0.18[b]	5.02	0.0004	0.0020
Mucispirillum	0.55±0.34[b]	2.22±0.66[a]	0.13±0.02[b]	5.02	0.0015	0.0025
Prevotellaceae_NK3B31_group	1.35±0.62[a]	0.07±0.05[b]	0.91±0.25[a]	4.81	0.0140	0.0149
Oscillibacter	0.46±0.10[b]	1.40±0.22[a]	0.35±0.04[b]	4.72	0.0007	0.0023
Roseburia	1.14±0.33[a]	0.42±0.05[b]	0.08±0.05[c]	4.72	0.0012	0.0024
Alloprevotella	0.63±0.14[b]	1.47±0.32[a]	0.44±0.13[b]	4.71	0.0142	0.0149
Colidextribacter	0.56±0.09[b]	1.50±0.19[a]	0.51±0.09[b]	4.69	0.0011	0.0024
Desulfovibrio	0.62±0.15[b]	1.27±0.22[a]	0.38±0.10[b]	4.65	0.0070	0.0085
unclassified Firmicutes	0.59±0.09[b]	1.42±0.17[a]	0.58±0.05[b]	4.63	0.0004	0.0020
Rikenella	0.32±0.14[b]	0.90±0.14[a]	0.16±0.03[b]	4.57	0.0023	0.0033
unclassified Peptococcaceae	0.23±0.08[b]	0.86±0.12[a]	0.20±0.03[b]	4.52	0.0011	0.0024
Anaerotruncus	0.15±0.05[b]	0.70±0.19[a]	0.10±0.02[b]	4.48	0.0012	0.0024
Rikenellaceae_RC9_gut_group	0.73±0.15[a]	0.85±0.16[a]	0.28±0.06[b]	4.45	0.0072	0.0085

[1] The significantly changed microbial genera were selected by the LefSe method with LDA score >3.0 and q value < 0.05.

[2] q values were calculated based on the *P* values with FDR correction.

SUPPLEMENTARY FIGURE

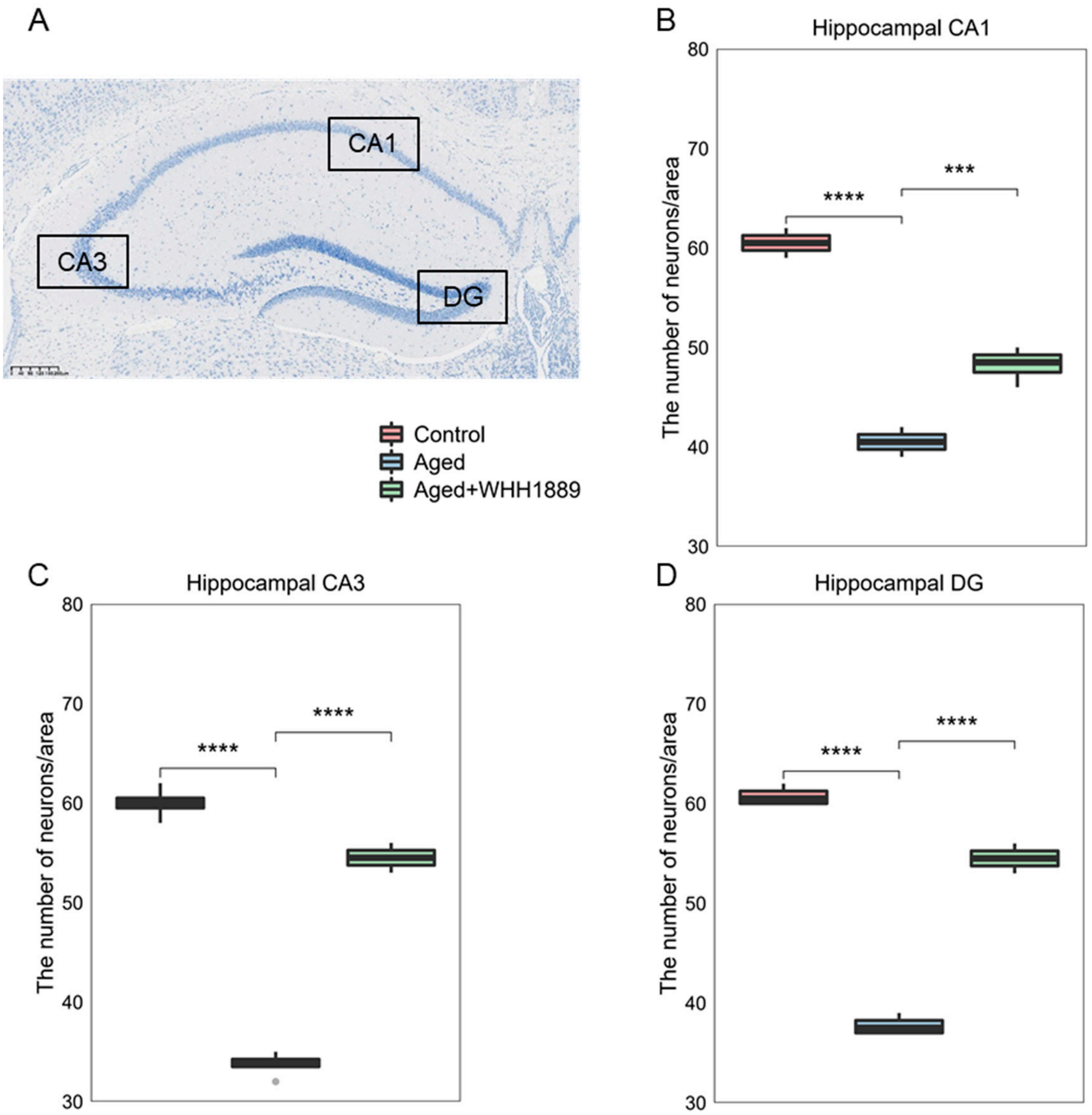

Figure S1. Effects of WHH1889 on the number of hippocampal neurons in aged mice. (A) The represnentative image of Nissl-stained hippocampal CA1, CA3 and DG areas in aged mice. The number of neuronal cells in the CA1 area (B), CA3 area (C), and DG area (D). The results are presented as mediums ± 95% CI (n=6). * *P*<0.05, ** *P*<0.01, *** *P*<0.001, **** *P*<0.0001.
